# Attenuated pulsatile transition to the cerebral vasculature during high‐intensity interval exercise in young healthy men

**DOI:** 10.1113/EP091119

**Published:** 2023-06-12

**Authors:** Jun Sugawara, Takeshi Hashimoto, Hayato Tsukamoto, Niels H. Secher, Shigehiko Ogoh

**Affiliations:** ^1^ Human Informatics and Interaction Research Institute National Institute of Advanced Industrial Science and Technology Tsukuba Japan; ^2^ Graduate School of Sport and Health Science Ritsumeikan University Shiga Japan; ^3^ Faculty of Sport Sciences Waseda University Saitama Japan; ^4^ Department of Anesthesia, Rigshospitalet, Institute for Clinical Medicine University of Copenhagen Denmark; ^5^ Graduate School of Engineering Toyo University Saitama Japan

**Keywords:** HIIT, transfer function analysis, Windkessel function

## Abstract

High‐intensity interval exercise (HIIE) is recommended because it provides favourable haemodynamic stimulation, but excessive haemodynamic fluctuations may be an adverse impact on the brain. We tested whether the cerebral vasculature is protected against systemic blood flow fluctuation during HIIE. Fourteen healthy men (age 24 ± 2 years) underwent four 4‐min exercises at 80–90% of maximal workload (*W*_max_) interspaced by 3‐min active rest at 50–60% *W*_max_. Transcranial Doppler measured middle cerebral artery blood velocity (CBV). Systemic haemodynamics (Modelflow) and aortic pressure (AoP, general transfer function) were estimated from an invasively recorded brachial arterial pressure waveform. Using transfer function analysis, gain and phase between AoP and CBV (0.39–10.0 Hz) were calculated. Stroke volume, aortic pulse pressure and pulsatile CBV increased during exercise (time effect: *P* < 0.0001 for all), but a time‐domain index of aortic–cerebral pulsatile transition (pulsatile CBV/pulsatile AoP) decreased throughout the exercise bouts (time effect: *P* < 0.0001). Furthermore, transfer function gain reduced, and phase increased throughout the exercise bouts (time effect: *P* < 0.0001 for both), suggesting the attenuation and delay of pulsatile transition. The cerebral vascular conductance index (mean CBV/mean arterial pressure; time effect: *P* = 0.296), an inverse index of cerebral vascular tone, did not change even though systemic vascular conductance increased during exercise (time effect: *P* < 0.0001). The arterial system to the cerebral vasculature may attenuate pulsatile transition during HIIE as a defence mechanism against pulsatile fluctuation for the cerebral vasculature.

## Abstract (highlights)

- **What is the central question of this study?**
  High‐intensity interval exercise (HIIE) is recommended for its favourable haemodynamic stimulation, but excessive haemodynamic fluctuations may stress the brain: is the cerebral vasculature protected against exaggerated systemic blood flow fluctuation during HIIE?
- **What is the main finding and its importance?**
  Time‐ and frequency‐domain indices of aortic–cerebral pulsatile transition were lowered during HIIE. The findings suggest that the arterial system to the cerebral vasculature may attenuate pulsatile transition during HIIE as a defence mechanism against pulsatile fluctuation for the cerebral vasculature.

## 1 INTRODUCTION

For both physical fitness and preparation of athletes, high‐intensity interval training (HIIT) with short alternating periods of high‐intensity exercise followed by brief recovery periods has drawn attention (Whitaker et al., 2020). Also, HIIT has been applied for rehabilitation for cardiovascular disease, given its capacity to potentiate metabolic, cardiopulmonary and systemic vascular adaptation (Weston et al., 2014). A systematic review and meta‐analysis showed that HIIT represents a potent stimulus in improving endothelial function and induces positive effects on cardiorespiratory fitness, cardiovascular disease risk factors and biomarkers associated with vascular function relative to moderate‐intensity continuous exercise training (Ramos et al., 2015). Similarly, HIIT has been applied for rehabilitation in patients with brain‐related pathology such as stroke (Boyne et al., 2013). It has been reported that a single bout of high‐intensity interval exercise (HIIE) increases the internal carotid arterial shear rate more than the equivalent volume of moderate‐intensity continuous exercise training, indicating that HIIT is more effective in improving endothelial function than continuous exercise (Ogoh et al., 2021).

During HIIE, stroke volume (SV) increases, eliciting excessive pressure and blood flow fluctuations that likely enhance shear stress and improve endothelial function (Calverley et al., 2020). Yet, since the brain is a highly metabolically active organ with low vascular resistance, it could be affected by excessive pressure and blood flow fluctuations (O'Rourke & Safar, 2005). In addition, HIIE elicits a rapid increase in systemic blood pressure. Despite hyperventilation‐induced vasoconstriction, Calverley et al. (2020) argued that unless these actions are countered by the shock‐absorbing effect of sympathetic activation or cerebral autoregulation (CA), HIIE could increase the risk of cerebral hyperperfusion injury predisposing to stroke or disruption of the blood–brain barrier. In this context, we demonstrated that the transfer function phase in the low‐frequency range (LF; 0.07−0.20 Hz) increases during HIIE, while the gain in the LF range does not change (Tsukamoto et al., 2019), indicating that dynamic CA is maintained. However, the shock‐absorbing function of the arterial system to the cerebral vasculature during HIIE, especially in the higher frequency range that cannot be modified via dynamic CA, is yet to be explored.

At rest, central elastic arteries receive fluctuating flow generated by intermittent ejection of blood from the left ventricle but pass blood on in a laminar stream through peripheral arterioles and capillaries (Nichols & McDonald, 2011). Considering the property of the arterial wall (i.e., distensibility), compared with the intracranial arteries, a larger role of the aorta and the extracranial (e.g., carotid and vertebral) arteries in buffering that pulsatile pattern could be expected. A recent study demonstrated that carotid artery cross‐sectional compliance increased and the β‐stiffness index decreased during moderate‐intensity aerobic exercise (Fico et al., 2022), suggesting the temporary increase in the Windkessel function of the carotid artery. However, the effects of HIIE on the Windkessel function of the aorta and the extracranial arteries near the brain remain unclear.

Arterial pressure is often measured in peripheral arteries such as the upper arm, forearm, or finger. Still, pulsatility is influenced by reflected waves and increased arterial stiffness (peripheral pulse amplification) (Nichols & McDonald, 2011). In other words, there is a discrepancy between the pulsatile fluctuations of arterial pressure in central and peripheral arteries, which is enhanced during dynamic exercise (Rowell et al., 1968). Because monitoring peripheral blood pressure, which has been most investigated, overestimates the pulsatile fluctuation of central arterial pressure during exercise, little is known about how central arterial pressure changes during HIIE and affects cerebral circulation.

Accordingly, we estimated the haemodynamic transition from the aorta to the cerebral vessels during HIIE alternating between high‐intensity exercise and active rest in young, healthy men. We hypothesized that the transfer function gain between aortic pressure (AoP) and middle cerebral artery blood velocity (CBV) decreases during HIIE.

## 2 METHODS

### 2.1 Ethical approval

All procedures conformed to the *Declaration of Helsinki* (Seventh revision, 64th Meeting, Fortaleza, 2013) and were approved by the ethics committee in Copenhagen (H‐15011242). All participants gave written informed consent before participation.

### 2.2 Subjects

Fourteen healthy men (mean ± SD; age, 24 ± 2 years; height, 181 ± 6 cm; weight, 77 ± 8 kg) participated in this study after written, informed consent. All subjects were non‐smokers and were free of any known neurological, cardiovascular or pulmonary disorders. Subjects were instructed to avoid strenuous physical activity for 24 h before each experimental session and asked to abstain from food (overnight fast), caffeine and alcohol for 12 h. Experiments were performed at 22−24°C.

### 2.3 Experimental procedure

Before the day of the experiment, individual maximal workload (*W*_max_) was determined to calculate the intensity required for the exercise protocols. The test began at 30 W for 3 min and thereafter increased by 15 W/min until the subject could not maintain a cadence of 60 rpm as indicated by a metronome and carefully checked by an examiner. The *W*_max_ was determined as the highest workload kept for ≥30 s. The mean duration of the *W*_max_ test was 18 ± 3 min. The mean *W*_max_ for all subjects was 245 ± 43 W. On the experiment day, subjects had a standard light meal (580 kcal) 2 h before the HIIE test. Subsequently, each subject underwent HIIE in a semi‐recumbent position with the upper body at a 40° inclination with a Krogh cycle ergometer attached to a bed. The subjects relaxed their legs on the bed at rest until the feet were strapped to the pedals of the cycle during exercise. HIIE included four 4 min of exercise at 80–90% of *W*_max_ interspaced by 3‐min active rest at 50–60% *W*_max_ that was conducted after a 5‐min warm‐up at 50−60% *W*_max_ (Hashimoto et al., 2018).

### 2.4 Measurements

A lead II electrocardiogram monitored heart rate (HR). CBV at the middle cerebral artery (MCA) was measured using transcranial Doppler ultrasonography (Compumedics, Singen, Germany) with a 2‐MHz probe placed over the temporal ultrasound window. An optimal signal‐to‐noise ratio was obtained at a depth of 48−60 mm, and the probe was fixed with an adjustable headband. A catheter (1.1 mm inner diameter, 20 gauge) was placed in the brachial artery of the non‐dominant arm for the determination of cardiovascular variables. A pressure transducer (Baxter Nederland, Utrecht, The Netherlands), zeroed at the right atrium level, was interfaced with a Dialogue 2000 monitor (IBC‐Danica, Copenhagen, Denmark). Electrocardiogram, CBV, and arterial pressure data were sampled at 1000 Hz and stored for offline analysis (Chart v5.2 and Powerlab; ADInstruments, Bella Vista, NSW, Australia). AoP was estimated from the brachial arterial pressure waveforms via a validated generalized transfer function technique (SphygmoCor, AtCor Medical, Sydney, Australia) (Sugawara et al., 2010, 2013). SV was estimated offline using the Modelflow method (Wesseling et al., 1993) (Beatscope; Finapres Medical Systems, Enschede, The Netherlands), which simulates aortic flow waveforms from an arterial pressure signal using a non‐linear, three‐element model of the aortic input impedance taking height, weight, age and sex into account. Cardiac output (CO) was SV × HR, and Modelflow estimates changes in SV and CO reliably during various experimental protocols, including dynamic exercise (Sugawara et al., 2003). Systemic vascular conductance was given by SVC = CO/mean arterial pressure (MAP), and cerebrovascular conductance index was given by CVCi = mean CBV/MAP, representing an inverse index of vascular tone. The ratio of pulsatile CBV to pulsatile AoP was obtained as a time‐domain index of aortic–cerebral pulsatile transition. The ratio of pulsatile CBV to mean CBV was calculated as a pulsatility index (PI). Arterial blood (1 ml) was drawn anaerobically at rest and after each high‐intensity exercise bout, and arterial carbon dioxide tension ($P_{\mathrm{aC}O_{2}}$) was measured by the blood gas analyser (ABL 800, Radiometer, Copenhagen, Denmark).

**FIGURE 1:**
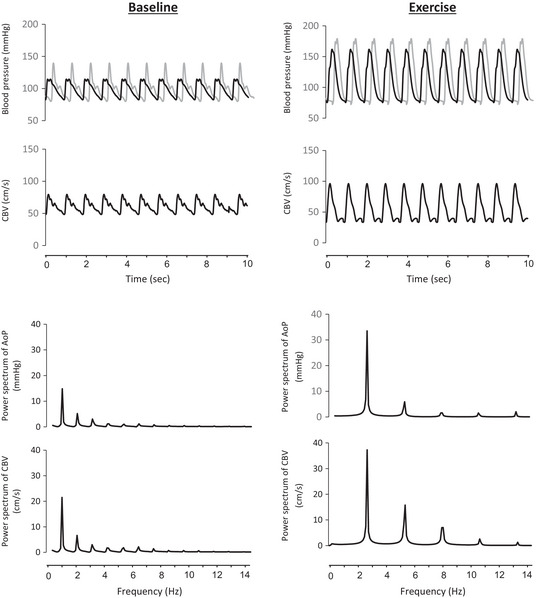
Representative data set showing time series and spectra of aortic pressure (AoP) and middle cerebral artery blood velocity (CBV) at rest and during exercise.

To evaluate frequency‐domain aortic–cerebral haemodynamic transition, HR‐related harmonic components of AoP and CBV were obtained by Fourier analysis (Sugawara et al., 2022, 2017) with a minor modification. For the calculation, changes in AoP were used as the ‘input’ signal and changes in CBV as the ‘output’ signal. Auto‐spectra and cross‐spectra of AoP and CBV were estimated using the Welch algorithm. The generalized transfer function technique using SphygmoCor can provide a valid amplitude of aortic pulse pressure but not the start of pressure upstroke (e.g., systolic foot). To minimize the influence of individual differences in the pulse transition time, the stable 10‐s time series of AoP and CBV waveforms captured from the last minute at each stage were adjusted to match the timing of both the first systolic foot of AoP and CBV (detected by the peak of the first derivative). By this procedure, we could evaluate the relative change in the time component of pulse transition from the aorta to the brain through the change in phase. Resampled data at 100 Hz were subdivided into 256‐point segments with a 50% overlap for spectral estimation. Each data segment was multiplied by a Hamming window before the periodogram estimation and averaged to result in a spectral resolution of 0.39 Hz. As shown in Figure 1, HR‐related harmonic peaks of spectral power in AoP and CBV were gradually attenuated at the integer multiple of HR frequency and levelled off after the fourth peak. Since the highest HR during HIIE was 176 beats/min (2.93 beat/s), the third HR‐related harmonic peak corresponded to 8.79 Hz (2.93 × 3). Therefore, in this study, we calculated transfer function gain, phase and coherence at the frequency range from 0.39–10.0 Hz, where most of the energy of signals was contained (Figure 1). In addition, to take the difference in HR at rest and during exercise into account, we focus on the responses of the first harmonic coherence, gain and phase.

### 2.5 Statistics

Data are presented as means and SD. After confirmation of normal distribution (via Shapiro–Wilk test), one‐way (time) or two‐way (frequency × time) repeated measures ANOVA was applied. Bonferroni's *post hoc* test was performed for significant main or interaction effects, and the statistical significance level was set at 5%.

## 3 RESULTS

The workload on the cycle ergometer was maintained throughout all sessions in 10 participants, whereas four participants requested the workload be reduced during the later session. The averaged power output of high‐intensity periods were 201 ± 36 (1st bout), 197 ± 32 (2nd bout), 196 ± 33 (3rd bout) and 196 ± 33 W (4th bout), respectively.

**FIGURE 2:**
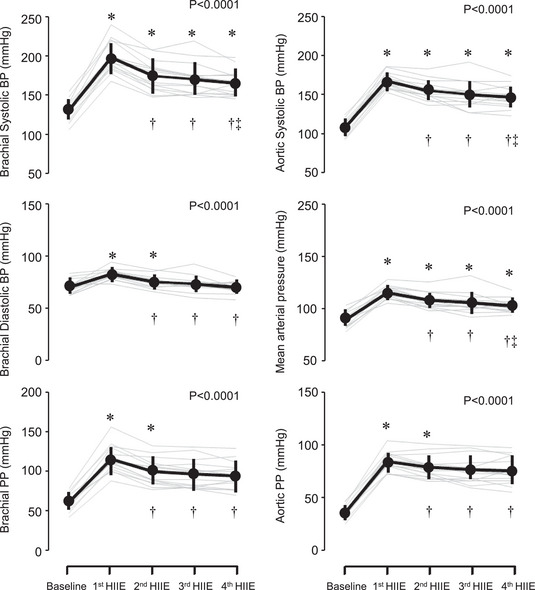
Responses of blood pressure (BP) and pulse pressure (PP) to the repetitive high‐intensity interval exercise (HIIE). Thick lines are mean and standard deviation (*n* = 14). Thin grey lines are individual responses. *, † and ‡ indicate significant differences from baseline, 1st HIIT and 2nd HIIT, respectively.

**FIGURE 3:**
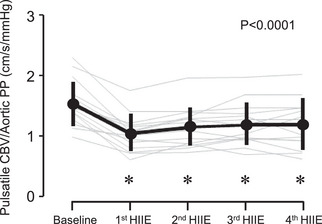
Responses of middle cerebral artery pulsatile blood velocity (pulsatile CBV)/aortic pulse pressure (PP) ratio to the repetitive high‐intensity interval exercise (HIIE). Thick lines are mean and standard deviation (*n* = 14). Thin grey lines are individual responses. *Significant difference from the baseline.

Systemic and cerebral haemodynamics and $P_{\mathrm{aC}O_{2}}$ are presented in Figure 2 and Table 1. Systolic blood pressure and pulse pressure elevations were the greatest at the first HIIE, and these hypertensive responses were gradually attenuated (Figure 2). HR, SV and CO increased, and $P_{\mathrm{aC}O_{2}}$ decreased during the exercise bouts. Systolic, mean and pulsatile CBV maintained significantly higher levels during the exercise than at rest, whereas diastolic CBV did not change significantly throughout the experimental protocol. PI increased throughout the exercise. PI at the second and third HIIE was significantly higher than the baseline (*P* = 0.04 and *P* = 0.02, respectively). Regarding responses of the index of vascular conductance to HIIE, SVC increased by three‐fold (*P* < 0.0001), whereas CVCi did not change significantly (*P* = 0.296). Pulsatile CBV/pulsatile AoP, an index of aortic–cerebral pulsatile transition, was reduced throughout the exercise bouts (*P* < 0.05 vs. baseline, Figure 3).

**TABLE 1:** Responses of systemic and cerebral haemodynamics and arterial carbon dioxide tension ($P_{\mathrm{aC}O_{2}}$) to high‐intensity interval exercise (HIIE).

	Baseline	1st HIIE	2nd HIIE	3rd HIIE	4th HIIT	*P*‐value (partial η^2^)
Heart rate (beat/min)	75 ± 9	158 ± 9*	166 ± 8*	169 ± 9*	176 ± 10*^†¶^	< 0.0001 (0.971)
Stroke volume (ml)	94 ± 19	127 ± 27*	135 ± 27*	137 ± 23*	138 ± 26*	< 0.0001 (0.653)
Cardiac output (l/min)	7.0 ± 1.5	18.2 ± 6.2*	21.2 ± 5.3*^†^	23.0 ± 3.6*^†^	24.1 ± 4.1*^†^	< 0.0001 (0.828)
SVC (l/min/mmHg)	0.08 ± 0.02	0.16 ± 0.06*	0.20 ± 0.05*^†^	0.22 ± 0.04*^†‡^	0.24 ± 0.03*^†‡^	< 0.0001 (0.841)
Systolic CBV (cm/s)	95 ± 19	132 ± 23*	132 ± 21*	132 ± 18*	128 ± 19*	< 0.0001 (0.734)
Diastolic CBV (cm/s)	42 ± 10	46 ± 10	45 ± 10	46 ± 9	44 ± 9	0.674 (0.043)
Mean CBV (cm/s)	59 ± 11	74 ± 12*	73 ± 12*	74 ± 11*	73 ± 15*	0.0009 (0.296)
Pulsatile CBV (cm/s)	53 ± 15	86 ± 20*	87 ± 20*	87 ± 20*	84 ± 20*	< 0.0001 (0.817)
Pulsatility index (%)	95.5 ± 22.1	120.3 ± 26.1	121.7 ± 32.9*	124.2 ± 32.6*	119.3 ± 27.9	0.011 (0.220)
CVCi (cm/s/mmHg)	0.66 ± 0.14	064 ± 0.12	0.68 ± 0.12	0.71 ± 0.12	0.73 ± 0.19	0.296 (0.089)
$P_{\mathrm{aC}O_{2}}$ (%)	40.4 ± 2.1	34.9 ± 3.3*	33.7 ± 3.1*	32.9 ± 3.6*^†^	31.4 ± 4.0*^†¶^	< 0.0001 (0.797)

Data are means and standard deviation (*n* = 14). *, †, ‡ and ¶ indicate significant differences from baseline, 1st HIIT, 2nd HIIT and 3rd HIIT, respectively. CBV, cerebral blood velocity; CVCi, cerebrovascular conductance index; SVC, systemic vascular conductance.

Table 2 summarizes averaged power spectrum at the first to third peaks. HR‐related harmonic peaks of spectral power in AoP and CBV were gradually attenuated at the integer multiple of HR frequency, irrespective of resting and exercise. The first harmonic peaks of spectral power in AoP and CBV increased significantly during HIIE from the baseline (at rest). The second harmonic peak of spectral power in CBV was significantly higher throughout the exercise than the baseline, whereas that of AoP was significantly higher at first and third HIIE than the baseline. The third harmonic peak of spectral power in AoP did not change significantly during the exercise bout, whereas that in CBV increased during the first HIIE and then returned toward the baseline after the second HIIE.

**TABLE 2:** Responses of aortic blood pressure and cerebral blood flow velocity power spectrum to high‐intensity interval exercise (HIIE).

	Baseline	1st HIIE	2nd HIIE	3rd HIIE	4th HIIT	*P*‐value (partial η^2^)
Power spectrum of aortic blood pressure (mmHg^2^)						
1st harmonic	12.2 ± 3.2	36.5 ± 4.3*	34.5 ± 5.5*	32.3 ± 8.2*^†^	34.1 ± 7.1*	Time: *P* < 0.0001 (0.807)
2nd harmonic ^a^	5.8 ± 1.9	10.6 ± 3.1*	9.0 ± 2.4	10.0 ± 2.4*	8.8 ± 2.2	Harmonics: *P* < 0.0001 (0.982)
3rd harmonic ^ab^	2.5 ± 0.7	3.9 ± 1.1	2.9 ± 1.0	2.5 ± 0.6	2.7 ± 0.8	Interaction: *P* < 0.0001 (0.769)
Power spectrum of cerebral blood velocity ((cm/s)^2^)						
1st harmonic	13.8 ± 5.4	30.5 ± 8.2*	30.9 ± 7.1*	32.3 ± 9.6*	31.8 ± 7.7*	Time: *P* < 0.0001 (0.650)
2nd harmonic ^a^	8.9 ± 2.7	13.3 ± 4.4*	15.5 ± 4.5*	19.5 ± 9.1*^†^	15.0 ± 4.1*‡	Harmonics: *P* < 0.0001 (0.941)
3rd harmonic ^ab^	5.5 ± 2.2	9.4 ± 2.5*	8.8 ± 2.5	8.4 ± 3.2	7.0 ± 2.2	Interaction: *P* < 0.0001 (0.607)

Data are means and standard deviation (*n* = 14). *, † and ‡ indicate significant differences from baseline, 1st HIIT and 3rd HIIT, respectively. ‘a’, ‘b’ and ‘c’ indicate significant differences versus 1st harmonics, 2nd harmonics and 3rd harmonics, respectively.

**FIGURE 4:**
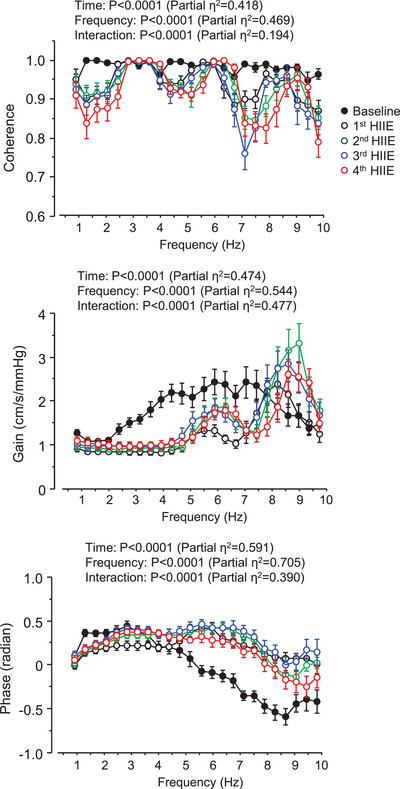
Transfer function coherence, gain and phase between aortic blood pressure and middle cerebral artery blood velocity (CBV) at the baseline (resting) and during the repetitive high‐intensity interval exercise (HIIE). Data are means and standard deviation (*n* = 14).

**FIGURE 5:**
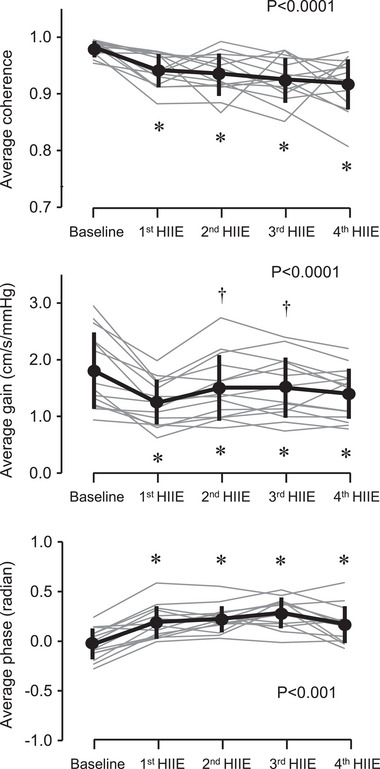
Exercise responses of average transfer function coherence, gain and phase between aortic blood pressure and middle cerebral artery blood velocity (CBV) in the frequency range 0.78–10 Hz. Thick lines are mean and standard deviation (*n* = 14). Thin grey lines are individual responses. * and † indicate significant differences from baseline and 1st HIIT, respectively.

**FIGURE 6:**
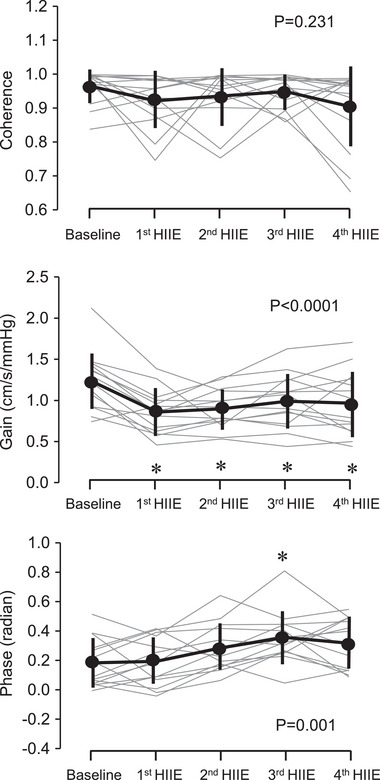
Exercise responses of transfer function coherence, gain and phase between aortic blood pressure and middle cerebral artery blood velocity (CBV) at the first harmonic of cardiac frequency. Thick lines are mean and standard deviation (*n* = 14). Thin grey lines are individual responses. * and † indicate significant differences from baseline and 1st HIIT, respectively.

Figure 4 depicts changes in the transfer function coherence, gain and phase between AoP and CBV in the frequency range 0.39–10 Hz. Averaged values in this frequency range are shown in Figure 5. Coherence was higher than 0.9, although coherence gradually decreased with repeated exercise (*P* < 0.0001). Gain reduced and phase increased throughout the exercise bouts (*P* < 0.0001 for both). Figure 6 shows coherence, gain and phase responses at the first harmonic of cardiac frequency. Coherence did not change with HIIE (*P* = 0.231). Gain decreased throughout the HIIE (*P* < 0.0001), whereas phase increased gradually (*P* < 0.0001) and was significantly higher at the third HIIE than the baseline (*P* = 0.005).

## 4 DISCUSSION

This study examined the response of the pulsatile transition in the arterial system from the aorta to the cerebral vessels during HIIE, alternating between high‐intensity exercise and active rest in young, healthy men. The main findings are that transfer function gain was reduced and phase was increased in the frequency range from 0.39 to 10.0 Hz throughout the exercise bouts. The transfer function gain at the first harmonic of cardiac frequency was significantly attenuated throughout the HIIE. The time‐domain index of aortic–cerebral pulsatile transition evaluated by pulsatile CBV/pulsatile AoP showed a similar result (e.g., significant reduction during the exercise bouts). Taken together, the arterial system from the aorta to the cerebral vasculature attenuates pulsatile transition during HIIE as a defence mechanism against pulsatile fluctuation for the cerebral vasculature.

Attenuated pulsatile cerebrovascular haemodynamics fluctuation and adequate cerebral perfusion are essential determinants of cerebrovascular health (O'Rourke & Safar, 2005). Using time‐resolved (cine) phase‐contrast magnetic resonance imaging reveals the coupling of the brain blood flow inside the skull over a cardiac cycle (Zhu et al., 2006). SV generated by the LV contraction propagates a systolic pulse wave inside a rigid skull with a fixed volume (Zhu et al., 2006). Excessive haemodynamic fluctuation is important for high blood flow and low‐resistance end‐organs (Mitchell, 2008; O'Rourke & Safar, 2005). Central elastic arteries (e.g., aorta and common carotid artery) effectively buffer cardiac ejection‐induced flow/pressure fluctuations (Nichols & McDonald, 2011). The high central arterial compliance in endurance athletes can accommodate endurance training‐related increases in SV and buffer the potential increase in pulsatility within the brain (Tomoto et al., 2018, 2015). Likely the Windkessel function of central arteries offsets the increase in the pulsatile component of cerebral perfusion induced by enhanced LV systolic function.

With the increase in SV during dynamic exercise to meet the oxygen demand, pulsatile blood flow, and arterial pressure fluctuations are enhanced. We previously reported that the transfer function gain in the low‐frequency range (LF; 0.07−0.20 Hz) did not change during HIIE (Tsukamoto et al., 2019), indicating the maintained dynamic CA which may stabilize cerebral perfusion during low‐frequency fluctuation in MAP (< 0.20 Hz). However, the shock‐absorbing function of the arterial system to the cerebral vasculature during HIIE, especially in the higher frequency range that cannot be modified by dynamic CA, remains unclear. Thus, we evaluated whether high‐frequency haemodynamic fluctuation is augmented and impacts the cerebral vasculature.

This is the first study to determine the haemodynamic transition to the middle cerebral artery during HIIE in a relatively higher frequency range (e.g., 0.39–10.0 Hz). Based on our previous studies (Sugawara et al., 2022, 2017), we analysed 10 s data segments in the present study. As shown in Figure 1, the target frequency range (0.78–10 Hz) included the first to third HR‐related haemodynamic oscillations even during HIIE in this study. We emphasized that high coherence provides confidence in the quality and reliability of data being interrogated. The average transfer function gain from AoP to CBV at the frequency range 0.39–10.0 Hz was reduced throughout the exercise bouts. Similar results were seen in these measures at the first harmonic and a time‐domain index of pulsatile transition (i.e., pulsatile CBV/pulsatile AoP). Furthermore, the pulsatile transmission was delayed as a significant increase in phase during HIIE. Our findings indicate that the arterial tree from the aorta to the middle cerebral artery effectively dampened haemodynamic pulsatility generated by cardiac ejection during exercise, which may protect against the breakdown of the blood–brain barrier.

We speculate on several mechanisms that could dampen MCA pulsatility. In the present study, CVCi remained unchanged during HIIE despite the substantial rise in SVC (a 3‐fold increase from the baseline). In the brain, unlike in skeletal muscle, the vasoconstrictive effect of the sympathetic system appears to outweigh metabolite‐induced vasodilatation (e.g., functional sympatholysis) (Calverley et al., 2020; Ogoh et al., 2015; Willie et al., 2014). Hyperventilation‐induced reduction in partial pressure of arterial carbon dioxide facilitates exercise‐related increases in cerebrovascular tone (Nybo & Nielsen, 2001; Nybo et al., 2002). Indeed, a significant reduction in $P_{\mathrm{aC}O_{2}}$ was observed during HIIE. Also, we previously observed the increase in external carotid artery blood flow and the concomitant decrease in internal carotid artery blood flow during dynamic high‐intensity cycling (Sato et al., 2011) and low‐intensity handgrip exercise (Hirasawa et al., 2016). Such heterogeneity and reciprocal regulation of intracranial vs. systemic (e.g., extracranial) blood flow may function as a protective mechanism for the brain against intracranial overperfusion that is usually associated with rapid increases in central blood volume and central arterial pulse (Ogoh et al., 2015).

This study involves several limitations. First, we studied only healthy young men, but there are no sex differences in the cerebral haemodynamic response to HIIT (Whitaker et al., 2022). However, the influence of female sex and reproductive hormones, primarily oestradiol, on cardiovascular control relevant to regulating blood pressure and cerebral blood flow is recognized (Barnes & Charkoudian, 2021). Age‐related arterial stiffening and endothelial dysfunction may also affect pulsatile transition during dynamic exercise, including HIIE. Second, aortic fluctuations in blood pressure were not measured by a catheter in the outlet from the heart. Although the reliability of this technique has been debated (Hope et al., 2008), invasive procedures depicted the accuracy of this technique during dynamic exercise (Sharman et al., 2006). Third, we measured CBV at MCA via TCD. Response of CBV measured with TCD might not reflect the change in actual cerebral blood flow. Although the MCA can dilate and constrict under certain conditions (Coverdale et al., 2014; Verbree et al., 2014), we do not know in the current study whether the MCA diameter is changing. Finally, some haemodynamic variables, such as SV, CO and SVC, were not measured directly. However, the Modelflow method could provide a reliable estimation of the relative change in these measures during high‐intensity dynamic exercise (e.g., 130% of ventilatory threshold) (Sugawara et al., 2003).

In conclusion, this study indicated that transfer function gain between AoP and CBV at the first harmonic of cardiac frequency was significantly attenuated during repetitive high‐intensity exercise bouts. A time‐domain index of aortic–cerebral pulsatile transition (pulsatile CBV/pulsatile AoP) also falls throughout HIIE bouts. These results suggest that the arterial system may attenuate pulsatile transition during HIIE as a defence mechanism against the large cerebral flow and pressure fluctuations.

## AUTHOR CONTRIBUTIONS

Hayato Tsukamoto, Takeshi Hashimoto and Shigehiko Ogoh conceived and designed the research. Hayato Tsukamoto, Takeshi Hashimoto, Niels H. Secher and Shigehiko Ogoh performed the experiments. Jun Sugawara and Hayato Tsukamoto analysed the data. Jun Sugawara, Hayato Tsukamoto, Takeshi Hashimoto, Niels H. Secher and Shigehiko Ogoh interpreted data. Jun Sugawara drafted the article. Niels H. Secher edited and revised the article. All authors have read and approved the final version of this manuscript and agree to be accountable for all aspects of the work in ensuring that questions related to the accuracy or integrity of any part of the work are appropriately investigated and resolved. All persons designated as authors qualify for authorship, and all those who qualify for authorship are listed.

## CONFLICT OF INTEREST

All authors disclose having no professional relationships with companies or manufacturers who will benefit from the results of the present study. The authors declare no conflict of interest.

## Supporting information


Statistical Summary Document

## Data Availability

The datasets generated and analysed during the current study are available from the corresponding author upon reasonable request.
